# Four equity considerations for the use of artificial intelligence in public health

**DOI:** 10.2471/BLT.19.237503

**Published:** 2020-02-25

**Authors:** Maxwell J Smith, Renata Axler, Sally Bean, Frank Rudzicz, James Shaw

**Affiliations:** a Faculty of Health Sciences, Arthur & Sonia Labatt Health Sciences Building, Western University, 1151 Richmond Street, London, N6A 5B9, Canada.; b Institute of Health Policy, Management, and Evaluation, University of Toronto, Toronto, Canada.; c Sunnybrook Health Sciences Centre, Toronto, Canada.; d Women’s College Hospital, Toronto, Canada.

New technologies can either improve or worsen health inequities.^1^ Innovative technologies involving artificial intelligence are no exception, particularly where they are adopted and implemented in health systems. Indeed, determining whether and how artificial intelligence might contribute to reducing or exacerbating health inequities has been identified as a priority research area by several stakeholders and by numerous ethics and policy guidance documents.^2^^–^^4^

Understanding the connection between health inequities and artificial intelligence should be a priority when deploying these technologies in public health. Since public health activities typically target populations instead of individuals and require collective action instead of individual intervention,^5^ introducing artificial intelligence technologies to support these activities may influence (either positively or negatively, intentionally or unintentionally) health inequities more than in other areas. As such, identifying the distinctive equity considerations and dimensions that might emerge in the public health context is critical.

However, doing so is not a straightforward task. First, we cannot simply look to past technological innovations to determine which health equity considerations or implications might arise with the use of artificial intelligence in public health because technological innovations and their diffusion in health systems each produce or interact with health inequities in novel ways.^1^ We may not be able to assume that the trends or pathways that create or prevent inequities will be the same when implementing artificial intelligence technologies as they are with other technological innovations. This limitation may be particularly challenging with artificial intelligence technologies given their use of big data and machine learning. Second, artificial intelligence represents a vast and sometimes contested area of study and application. Here we define artificial intelligence as a branch of computer science that explores the ability of computers to imitate aspects of intelligent human behaviour, such as problem-solving, reasoning and recognition.^2^ Technologies that are supported by artificial intelligence are therefore numerous, and include natural language processing, object recognition and reinforcement learning, among others. The ways in which these technologies might be deployed in public health are equally numerous, including digital disease surveillance, machine learning to predict incidences of noncommunicable diseases, and others. Finally, given that health inequities are often defined as differences in health that are unjust, even what should be counted as health inequities and what it means to achieve health equity may differ according to the nature of the new technology, how it is or has been integrated into health systems and our judgements about its interaction with the public’s health.^6^

As a result, before research or health system interventions in this area are developed or implemented, we should first seek to conceptually map the unique ways in which inequities might manifest when artificial intelligence is implemented or used in public health. Indeed, important work examining the unique equity dimensions associated with specific artificial intelligence technologies in this area has begun.^7^ Yet, we posit that there are general equity considerations and dimensions that can be identified and used as starting points for the reflection of equitable artificial intelligence in public health, and that it would be of benefit for the field to have these identified and enumerated. We will briefly describe four key equity considerations and dimensions and conclude by discussing how they can be used as starting points to further understand and enhance the equitable deployment of artificial intelligence in public health.

## Core aims

Before describing the four key equity considerations for artificial intelligence in public health, it is important to note two core aims that should be adopted given the prospect of deploying artificial intelligence technologies in the public health context. First, we should design and implement artificial intelligence technologies such that they do not create, sustain or exacerbate health inequities. This aim can be viewed as our negative aim, given its objective of preventing these new technologies from creating a situation that is worse than present. Second, given the promise that artificial intelligence holds for public health,^8^ we should design and implement artificial intelligence technologies such that they actively work to redress or eliminate health inequities, or otherwise promote health equity. This aim can be viewed as our positive aim, given its objective to leverage the opportunities posed by these technologies to create a situation that is better off than present. These two aims cut across each of the considerations described below such that both positive and negative actions can and should be taken to address them.

## Four equity considerations

### The digital divide

Perhaps the most obvious implications of implementing or relying upon new technologies in public health is the risk of unequal access to such technologies, inequalities in the opportunity to benefit from such technologies and inequalities in the burdens generated by such technologies.^9^ This digital divide between those who may or may not benefit from these technologies may manifest or become exacerbated between population groups (for example, groups with different socioeconomic status, geographic location, age, abilities, disabilities, and others), but also between researchers, public and private sectors, and even health systems. A divide may also emerge between those who actively choose to use or benefit from artificial intelligence technologies (such as wearable devices) and those who actively choose not to, for example for privacy reasons. This consideration raises the question: how does the use of artificial intelligence in public health reinforce or remediate the gap between those who may benefit from public health (including its data and interventions) and those who do not?

### Algorithmic bias and values

Artificial intelligence systems must be programmed or trained with certain data that might be biased and will invariably reflect value judgements, for example, what it means for an algorithm to be fair, such as producing fair outcomes.^10^ These value judgements have the capacity to create, sustain or exacerbate health inequities.^11^ For example, applying machine learning to human language or text data for public health purposes could result in human-like semantic biases, including those that are discriminatory towards race or gender.^11^ This consideration raises the question: what conscious or unconscious biases and/or value judgements exist in our artificial intelligence systems, including in the ways we train those systems?

### Plurality of values across systems

If due attention is paid to the sorts of biases and value judgements that inform our artificial intelligence approaches and the training of these systems, it is likely that different values will manifest in these technologies across health systems, for example between local, provincial, territorial, state, national, and international systems, depending on cultural or societal norms and values. The possibility exists that the explicit identification of values for artificial intelligence technologies adopted in public health systems will lead to health technologies, health interventions or perhaps entire health systems that will tend to produce unique outputs or outcomes according to those values or assumptions. This result may in turn create differences in outcomes between health systems that are attributable, at least in part, to the many values and assumptions that exist within the artificial intelligence technologies used within those systems – which may constitute a source of health inequities. This consideration raises the question: to what extent do the explicit or tacit values and assumptions that inform artificial intelligence technologies in public health cohere across technologies, interventions and systems? Where different values and assumptions lead to health inequalities, should this be considered inequitable?

### Fair decision-making procedures

Given the apparent need to explicitly identify the values and assumptions that inform artificial intelligence systems and the training of those systems, in a pluralist society reaching consensus about what those values and assumptions ought to look like might be unlikely. In addition, reaching consensus on what equitable outcomes from artificial intelligence in public health should look like might also be challenging. In the absence of substantive agreement on these questions, we might instead install fair and inclusive processes for the design and use of artificial intelligence in public health. Such processes may include following procedural principles like transparency and accountability, engaging underrepresented population groups or those otherwise least likely to be advantaged by artificial intelligence technologies in decision-making, or prioritizing the needs of the least advantaged in the design and implementation of such technologies.^12^ This consideration raises the question: what should fair processes for the development and implementation of artificial intelligence technologies and approaches look like, and how should diverse populations be engaged in designing them?

## Conclusion

The particularities of specific artificial intelligence technologies and approaches, in addition to the contexts in which they are deployed in public health surveillance, research, interventions or decision-making, will nuance each of the considerations and dimensions outlined above. As such, these considerations and dimensions cannot necessarily provide a roadmap to account for or address concerns of equity that may be present in every use of artificial intelligence in public health. Rather, we hope that they will serve as a starting point for the promotion of equitable artificial intelligence in this area. The distinctive equity considerations or challenges that will surface when such technologies or approaches are used in public health may be experienced only by particular stakeholders or communities, in particular contexts or under certain circumstances. As such, efforts to further map and understand these equity considerations ought to be accomplished in such a way that captures the multiple perspectives that reflect the diverse populations who will ultimately be impacted by artificial intelligence approaches used in public health practice, policy and research.

## References

[1] Weiss D, Rydland HT, Øversveen E, Jensen MR, Solhaug S, Krokstad S. Innovative technologies and social inequalities in health: a scoping review of the literature. PLoS One. 2018 4 3;13(4):e0195447. 10.1371/journal.pone.0195447 29614114 PMC5882163

[2] Ethical, social, and political challenges of artificial intelligence in health. London: Future Advocacy with the Wellcome Trust; 2019. Available from: https://wellcome.ac.uk/sites/default/files/ai-in-health-ethical-social-political-challenges.pdf [cited 2019 May 11].

[3] Asilomar AI principles. Cambridge: Future Life Institute; 2017. Available from: https://futureoflife.org/ai-principles/?cn-reloaded=1 [cited 2019 May 11].

[4] Ethically aligned design: a vision for prioritizing human well-being with autonomous and intelligent systems. Piscataway: Institute of Electrical and Electronics Engineers; 2019. Available from: https://standards.ieee.org/content/dam/ieee-standards/standards/web/documents/other/ead_v2.pdf [cited 2015 May 11].

[5] Dawson A, Verweij M. Public health ethics: a manifesto. Public Health Ethics. 2008;1(1):1–2. 10.1093/phe/phn009

[6] Smith MJ. Health equity in public health: clarifying our commitment. Public Health Ethics. 2015;8(2):173–84. 10.1093/phe/phu042

[7] Floridi L, Cowls J, Beltrametti M, Chatila R, Chazerand P, Dignum V, et al. AI4People—An ethical framework for a good AI society: opportunities, risks, principles, and recommendations. Minds Mach (Dordr). 2018;28(4):689–707. 10.1007/s11023-018-9482-5 30930541 PMC6404626

[8] Wahl B, Cossy-Gantner A, Germann S, Schwalbe NR. Artificial intelligence (AI) and global health: how can AI contribute to health in resource-poor settings? BMJ Glob Health. 2018 8 29;3(4):e000798. 10.1136/bmjgh-2018-000798 30233828 PMC6135465

[9] Norris P. Digital divide: civic engagement, information poverty, and the Internet worldwide. New York: Cambridge University Press; 2001 10.1017/CBO9781139164887

[10] Friedler SA, Scheidegger C, Venkatasubramanian S. On the (im)possibility of fairness [preprint server]. Ithaca: Cornell University; 2016 Available from: https://arxiv.org/pdf/1609.07236.pdf [cited 2020 Feb 17].

[11] Caliskan A, Bryson JJ, Narayanan A. Semantics derived automatically from language corpora contain human-like biases. Science. 2017 4 14;356(6334):183–6. 10.1126/science.aal4230 28408601

[12] Hogan-Doran D. Computer says “no”: automation, algorithms and artificial intelligence in government decision-making. J Judicial Comm N S W. 2017;13(3):345–82.

